# Multipolar spatial electric field modulation for freeform electroactive hydrogel actuation

**DOI:** 10.1038/s41598-020-59318-3

**Published:** 2020-02-12

**Authors:** Moon-Young Choi, Yerin Shin, Hu Seung Lee, So Yeon Kim, Jun-Hee Na

**Affiliations:** 1 Department of Electrical, Electronics, and Communication Engineering Education, Chungnam National University, Daejeon, 34134 Republic of Korea; 2 Department of Convergence System Engineering, Chungnam National University, Daejeon, 34134 Republic of Korea; 3 Graduate School of Energy Science and Technology, Chungnam National University, Daejeon, 34134 Republic of Korea; 4 Department of Mechanical and Material Engineering Education, Chungnam National University, Daejeon, 34134 Republic of Korea; 5 Department of Chemical Engineering Education, Chungnam National University, Daejeon, 34134 Republic of Korea

**Keywords:** Gels and hydrogels, Polymers, Actuators, Computational methods

## Abstract

Electroactive hydrogels that exhibit large deformation in response to an electric field have received significant attention as a potential actuating material for soft actuators and artificial muscle. However, their mechanical actuation has been limited in simple bending or folding due to uniform electric field modulation. To implement complex movements, a pre-program, such as a hinge and a multilayer pattern, is usually required for the actuator in advance. Here, we propose a reprogrammable actuating method and sophisticated manipulation by using multipolar three-dimensional electric field modulation without pre-program. Through the multipolar spatial electric field modulator, which controls the polarity/intensity of the electric field in three-dimensions, complex three-dimensional (3D) actuation of single hydrogels are achieved. Also, air bubbles generated during operation in the conventional horizontal configuration are not an issue in the proposed new vertical configuration. We demonstrate soft robotic actuators, including basic bending mechanics in terms of controllability and reliability, and several 3D shapes having positive and negative curvature can easily be achieved in a single sheet, paving the way for continuously reconfigurable materials.

## Introduction

Shape transformations are driven by inhomogeneous in-plane deformation of thin elastic sheets provide one of the efficient ways to reconfigurable three-dimensional (3D) structures^1–4^. Thus far, most previous results have focused on sheets that can access only a single trajectory from flat to a programmed shape, which pre-defined on the material^5–9^. Integrating a responsive material^10^ or geometric structure design^11^ that can be driven independently by two or more stimuli gives access to many pre-programmed shapes, but the difficulty of making and controlling these pattern sheets increases rapidly with the number of orthogonal controllable elements. Alternatively, re-writable hydrogels^12,13^ or shape memory polymers^14–16^ or liquid crystalline polymers^17,18^ have been demonstrated to form several shapes from a single sheet. However, these approaches need many times for re-defining patterns. Electroactive materials are of great interest in this respect, as they should allow for continuous reprogramming into an arbitrary number of shapes defined by polarity and intensity of the applied electric field^11,19^. Especially, hydrogel-based actuators can be exchanged chemical components or energy for aqueous mediums and operate by the reaction of gel network response^20–22^. Also, the hydrogel network can be pre-patterned for directional operation in response to the environment. Stimulating the movement of the hydrogel using the external electric field is appealing because of reliable control of signal strength and direction. This stimulus requires only ions in the external solution to induce operation. An electric field applied to a polyelectrolyte network locked in an electrolyte solution causes an asymmetrical dispersion of the ions, creating an osmotic pressure difference that was swelling and deforms the gel. These physical and chemical processes are similar to the transfer ion exchange between the cell membrane and its environment^23,24^.

While numerous routes have been developed to drive self-actuating of polyelectrolyte hydrogel by using an electric field, the complexity of the structures achieved is yet to match even that of defining *in-situ* two-directional bendings in a single sheet.

Indeed, most of the work to date has focused on simple bending for microfluidic and drug delivery that are formed either by introducing uniform curvature in a patterned two-dimensional (2D) sheet^19,25–27^ or by moving a pattern of the worm-like cantilever that controls bending speed^11,28,29^. Notable exceptions include approaches developed by Dickey and co-workers^10^ and Lee and co-workers^11^ where the bi-directional movement is controlled with two-type of hydrogel and thickness varying of a hydrogel structure on its actuation. Although it has been proven that it uses a single-direction electric field, it has not yet been able to carry out complex deformation without external components but at the same time, multi-directional operation.

**Figure 1:**
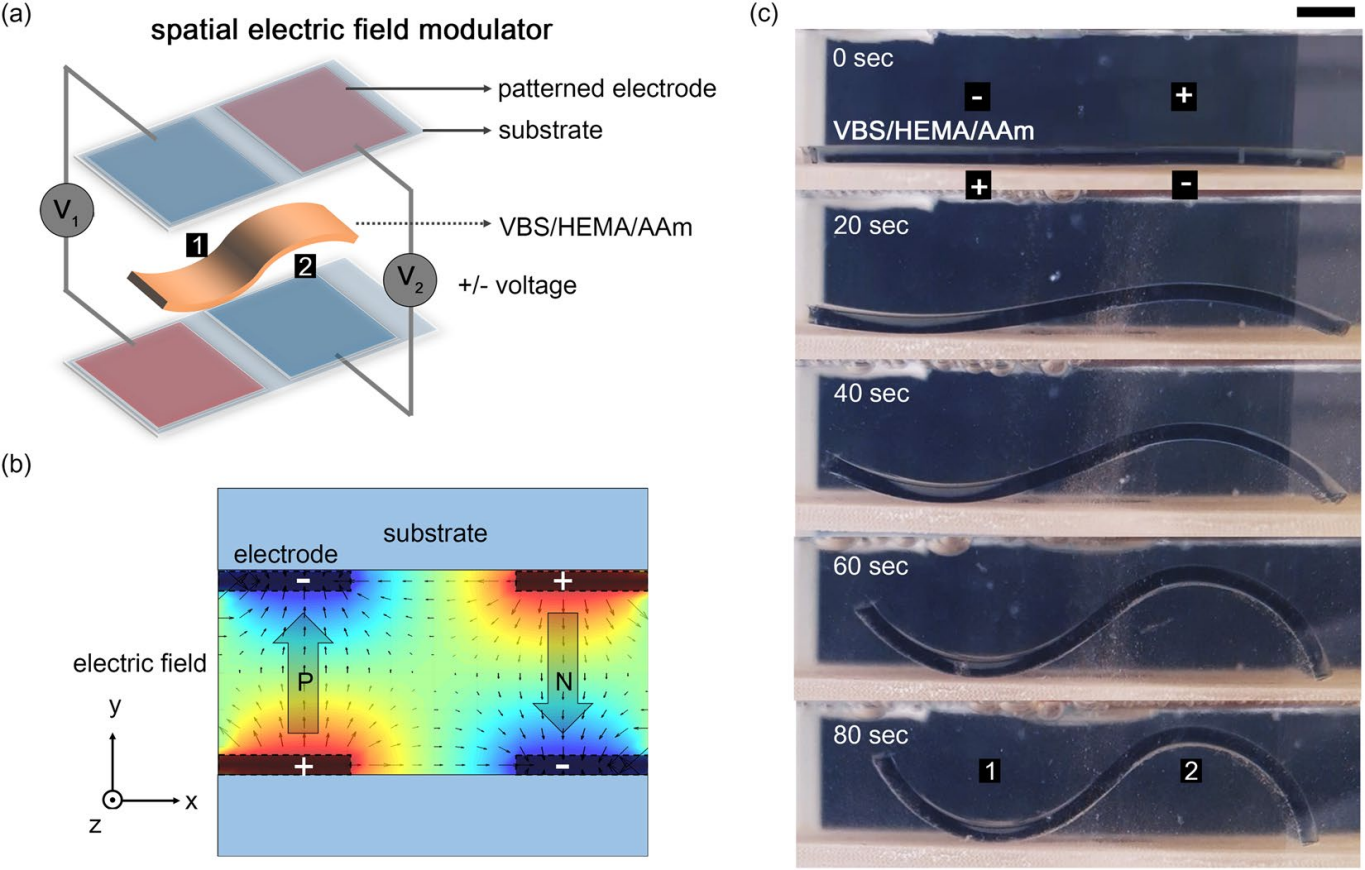
Strategies for electroactive hydrogel morphing with the multipolar spatial electric field. (**a**) Schematic diagram of the positive and negative curvatures bending for hydrogel as a function of the separated electric field with a vertical electrode configuration. (**b**) The two-dimensional electric field distribution of the multipolar electrode. The direction of the electric field was controlled by controlling the polarity of the voltage to each electrode, and P and N depict the electric field direction. (**c**) Sequential images of hydrogel having bidirectional bending. The part-1 and part-2 indicate positive (toward top electrode) and negative (toward bottom electrode) curvature, respectively. The scale bar corresponds to 3 mm.

Here, to achieve arbitrary and fully reprogrammable soft actuators based on local deformation within a single hydrogel sheet, we take advantage of spatial electric field modulation due to the patterned electrode, coupled with an electroactive hydrogel of sodium 4-vinylbenzene sulfonate/2-hydroxyethyl methacrylate/acrylamide (VBS/HEMA/AAm) to drive bi-directional deformation as shown in Fig. 1(a).

## Results

### Strategies for electroactive hydrogel morphing with the multipolar spatial electric field

As many previous studies in many research groups show, electroactive hydrogel, which reacts to the electric field, has a swell-deswell characteristic depending on the intensity or polarity of the electric field, which can be used to implement shape-morphing^1,25,30^. Figure 1 shows our concept of a system to spatial electric field modulation of the hydrogel. The patterned electrodes located on the upper and lower substrates, and each electrode could be operated independently. To apply an electric field in a vertical configuration, each pair of electrodes of the top and bottom substrate appropriately aligned. The patterned VBS/HEMA/AAm, which located between the top and bottom substrate, reacts to the electric field and swell by asymmetrical dispersion of the ions. Figure 1(b) shows the two-dimensional electric field distribution when a multipolar voltage is applied to an electrode with two pairs of electrodes arranged vertically, and the electric field can be controlled spatially. The labeled parts 1 and 2 indicate that VBS/HEMA/AAm banded in the opposite direction by an electric field in different polarity as a function of time. When this vertical configuration is used, a simple electrode pattern can be used to implement multiple deformation properties in a single sample without hinge patterns or pre-program, as shown in Fig. 1(c). Also, the vertical configuration and patterned electrodes were able to increase the degree of freedom in terms of shape-deformation compare to a conventional horizontal configuration^11,12,19,30^ having a single pair of electrodes. In addition, the conventional driving method of horizontal electrode configuration has difficulty in driving for a long time because air bubbles generated by electrolysis stick to the sample, but our vertical configuration method would be more free from this issue. We conducted electro-actuation experiments of hydrogel networks of equimolar composition equilibrated in 0.025 M NaCl and average swelling ratio of 14.3 was obtained. An electro-active copolymer of VBS/HEMA/AAm was patterned 30 mm by 10 mm rectangular gel bends toward the cathode in an aqueous medium. Also, the CO_2_ Laser cutting machine was used for electroactive hydrogel patterning as well as substrates fabrication.

### Numerical simulations of multipolar spatial electric field modulation

**Figure 2:**
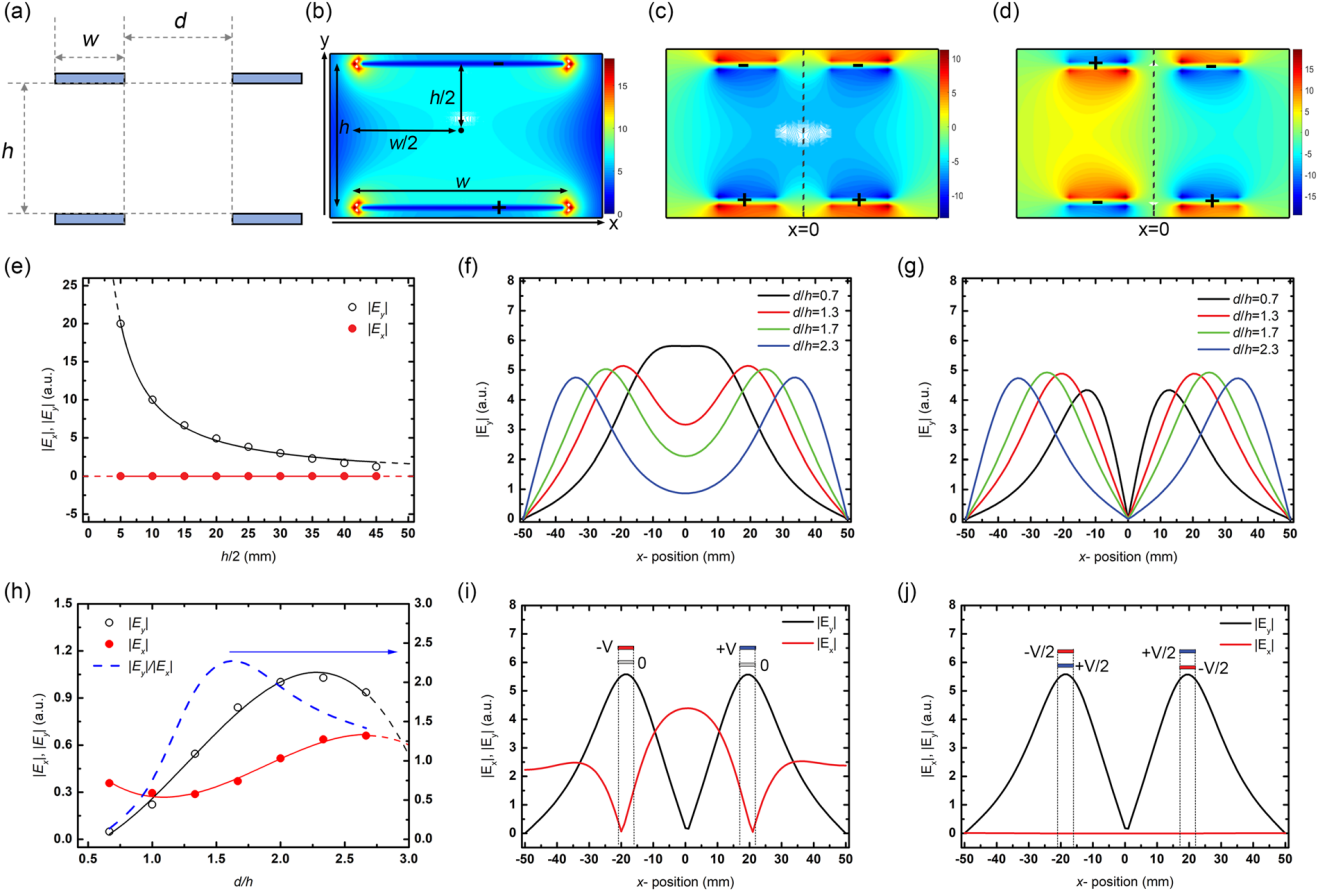
Numerical simulations of multipolar spatial electric field modulation. (**a**) The dimension of electrodes in a vertical configuration. Here, *w*, *d*, and *h* depict the width of the electrode, the horizontal distance between patterned electrodes, and the vertical distance of the electrode pairs, respectively. (**b**) The electric field distribution of a single pair (*d* = 0) of the electrode geometry. The total electric field distribution in patterned electrode with (**c**) the same polarity and (**d**) different polarity in the electrode pairs vertically. (**e**) The orthogonal electric field of |*E*_x_| and |*E*_y_| as a function of *h*/2 in case of single pair (*d* = 0) of the electrode configuration. The spatial variations of the active electric field of |*E*_y_| with (**f**) the same polarity of the electric field configuration and with (**g**) different polarity of one in cases of *d*/*h* = 0.7, 1.3, 1.7, and 2.3. (**h**) The spatial variations of the active electric field of |*E*_*y*_| (black) and |*E*_*x*_| (red) as a function of *d*/*h* in patterned electrode configuration. The |*E*_y_|/|*E*_x_| (blue dash), which significant in vertical electrode configuration, has an optimum value in *d*/*h* of ~1.6. The orthogonal electric field of |*E*_x_| and |*E*_y_| as a function of *x*-position (**i**) when bottom electrodes are grounded and (**j**) when voltages are applied in half (±v/2) to the top and bottom electrodes.

To calculate the distribution of the electric field in two-dimensional space, Fig. 2 shows the multipolar spatial electric field characteristics according to the vertical electrode configuration. The simulation of the spatial electric field was carried out with the horizontal spacing (*d*) between the patterned electrodes and the vertical distance (*h*) of between the electrodes as variables in the vertical electrode configuration. Here, *w* is the width of the patterned electrode. The electric field characteristics, according to the ratio of *d*/*h*, are observed as the critical parameter because the correlation between the vertical and horizontal electric fields is a significant factor in generating the spatial electric field.

Figure 2(b) shows the electric field distribution of a single pair of the electrode (*d* = 0) in a vertical configuration. As shown in the distribution of electric fields along the *y*-axis, one can observe that the intensity of electric fields decreases as the distance from electrodes. In other words, the region close to electrodes has a larger vertical electric field. The variation of the *y*-axis electric field, |*E*_*y*_|, is noticeable as a function of *h* while the *x*-axis electric field, |*E*_*x*_|, is negligible, as shown in Fig. 2(e). It was found that the |*E*_*y*_| decreased as a function of *h*/2, and it is necessary to reduce the *h* in order to cause a large deformation with the larger |*E*_*y*_| while it could be a limit to the out-of-plane deformation of the sheet.

Next, we consider the distribution of electric fields when two pairs of patterned electrodes are having with the same polarity (Fig. 2(c)) and with the different polarity of applied voltage (Fig. 2(d)). Applying the same voltage to the upper (or lower) electrode will produce the same polarity electric field distribution overall, as shown in Fig. 2(c). This field distribution can also be seen in Fig. 2(f), which varies the *d* (The *h* was fixed). In cases of *d*/*h *< 1.7, the distinction of the |*E*_*y*_| by adjacent electrode pairs is ambiguous, and it is not suitable for electroactive actuators, which are intended to induce deformation independently. Therefore, *d*/*h* should be at least 1.7 for precise electric field modulation in case of applying the same polarity, and it is challenging to control a precise electric field modulation in the same polarity.

When a reverse voltage is applied to patterned electrode pairs, a spatially separated electric field can be generated, as shown in Fig. 2(d). This field distribution was informed that with importance to the spatial field control that component of vertical electric field control is possible independently of the polarity of the applied voltage. Figure 2(g) shows the distribution of the |*E*_*y*_| according to the *d*/*h*, which shows the vertical electric field is ~0 V/m at the middle region (at *x* = 0). In other words, it was confirmed that different polarity electric fields could be obtained between the two adjacent electrode pairs, and spatial electric field control is possible through the electrode pattern regardless of *d*. Even the vulnerable regions of *x* = 0 in terms of the |*E*_*y*_|, the |*E*_*y*_| could be dominant compared to |*E*_*x*_| in case of *d* > *h*. To optimized multipolar electric field modulator, the electric field of |*E*_y_| and |*E*_x_| were verified in terms of *d*/*h*. |*E*_x_| is a component that hinders proper deformation of the hydrogel in the vertical electrode structure, and |*E*_y_| is a component that induces out-of-plane deformation, properly. Therefore, it is essential to find the electrode structure to realize the optimum modulation, and it can be confirmed that *d*/*h* ~ 1.6 is optimal, as shown in Fig. 2(h).

In addition, we examined the application of voltage to minimize |*E*_*x*_|. Both Fig. 2(i,j) apply +V and −V to two pairs of electrodes. Figure 2(i) set the lower electrode to ground (V = 0) and Fig. 2(j) were applied to the upper and lower electrodes as ±v/2. As can be seen from the simulation, even if the same voltage is applied, a method that can minimize the electric field with adjacent electrodes in the *x*-axis direction can be expected to be advantageous for driving.

### Electroactive hydrogel bending mechanics

**Figure 3:**
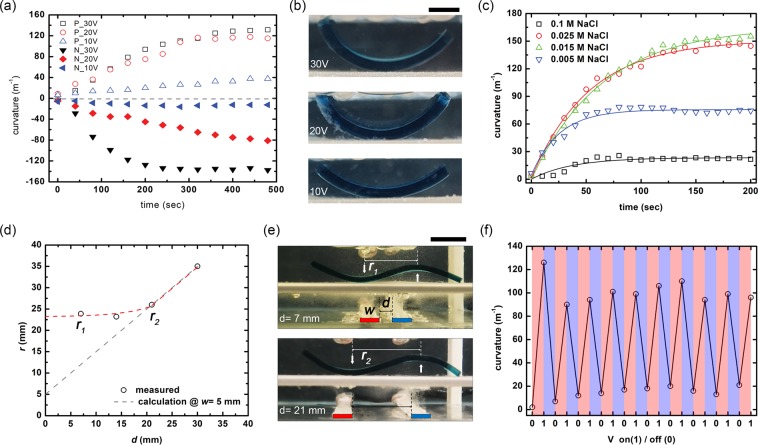
Electroactive hydrogel bending mechanics. (**a**) The curvature is plotted against the time of the applied electric field for three different values of voltage. Positive curvature indicates bending toward the upper electrode, and negative curvature indicates bending toward the lower electrode. (**b**) Images in a constant applied an electric field of 30, 20, and 10 V (Scale bar is 5 mm). (**c**) The bending curvatures in cases of 0.005, 0.015, 0.025, and 0.01 M NaCl. (**d**) The effective distance between the positive and negative curvatures according to the periodicity of the electric field is measured and plotted for the cantilever in (**e**). Here, *r* depicts the distance between two bending and the scale bar is 10 mm. (**f**) The curvature of the bend is found to be reproducible through multiple cycles of voltage on and off, as plotted for the single bending cantilever in (**b**).

Based on the above numerical calculation results, we observed the bending mechanics of VBS/HEMA/AAm in the vertical electric field environment. Figure 3(a) shows the curvature varying by the applied voltage to 30, 20, and 10 V on the bottom electrode while holding the ground (0 V) on the bottom electrode. The curvature of each hydrogel increases as a function of time, and the higher the applied voltage, the faster bend. Here, all the voltage conditions are processed into new VBS/HEMA/AAm samples, and each sample being dimension 30 by 10 mm and 1.8 mm thick (see in Supporting Information Fig. S3(a)). The image of the VBS/HEMA/AAm copolymer according to the applied voltage conditions and is an observation after 500 sec with the voltage of 30, 20, and 10 V applying as shown in Fig. 3(b). To correlate the direction of the electric field, we also observed the characteristics of the applied voltage according to the polarity of the electric field. Here, P and N indicate positive and negative curvature, respectively. Both conditions of P and N have been observed to increase the curvature as the magnitude of the applied voltage increases, and it has been confirmed that the direction of bending can also be controlled in the polarity of the electric field. Figure 3(c) shows the effect of NaCl concentration in cases of 0.005, 0.015, 0.025, and 0.01 M, in terms of the bending mechanics. The small bending curvatures occur because mobile ions are not enough to induce significant osmotic pressure due to the lower NaCl concentrations. It was also confirmed that a sufficient amount of NaCl interfered with the bending mechanics, and we set the optimum concentration of NaCl to 0.025 M in terms of bending characteristic^11,31^.

The numerical calculation shows that voltages of different polarities can achieve positive and negative curvature in a single sample as shown in Fig. 2. Moreover, in the simulation, the periodicity of the electric field can be controlled according to the distance between adjacent electrodes, and accordingly, the deformation period (*r*) of the hydrogel cantilever can be linearly controlled. However, the experimental results (Fig. 3(d,e)) show that the *r* is limited as ~23 mm even keep decreasing the electrode gap (*d*). The deformation of the hydrogel generated by the electric field and the mechanical properties of the hydrogel were found to be conflicting, and thus the minimum area necessary for the deformation was present, which can be defined as the resolution. Probably, this actuation resolution varies depending on the mechanical properties of the hydrogel used and the electric field condition. Here, the vertical distance (*h*) of electrodes and width (*w*) of the electrode are set to 30 and 5 mm, respectively.

Using the bending of VBS/HEMA/AAm copolymer to guide our choices of driving conditions, we next consider how the bending stability according to the applied electric field. Based on the bending mechanics, it was noticed that the maximum curvature of copolymer increases as voltage increases. However, there is a limit to stability concerning voltage conditions. When operated for a long time at each driving voltage, the bending curvature is increased, but the operation is not stable after a specific point of time. Figure 3(f) shows the curvature of bend is found to be reproducible through multiple cycles of voltage on and off. Even the initial curvature was reduced by more than 20% after the first drive was performed at the 20 V, the bending characteristics were stably obtained after 10 cycles and each cycle was run at 30 minute intervals. Here, 0 and 1 mean electric field OFF and ON, and the electric field is 20 V. We suspect here that under the relatively higher voltage conditions, the VBS/HEMA/AAm copolymer is subjected to irreversible deformation. Previously, de Gennes and coworkers show that the soft gels suffer from certain defects when they give large deformations due to the certain microscale fractures and provide systems with a short lifetime^32–34^. The extensive electric field driving achieves relatively high curvature can be obtained, but not suitable for long-term driving stability.

### Movements of a starfish

**Figure 4:**
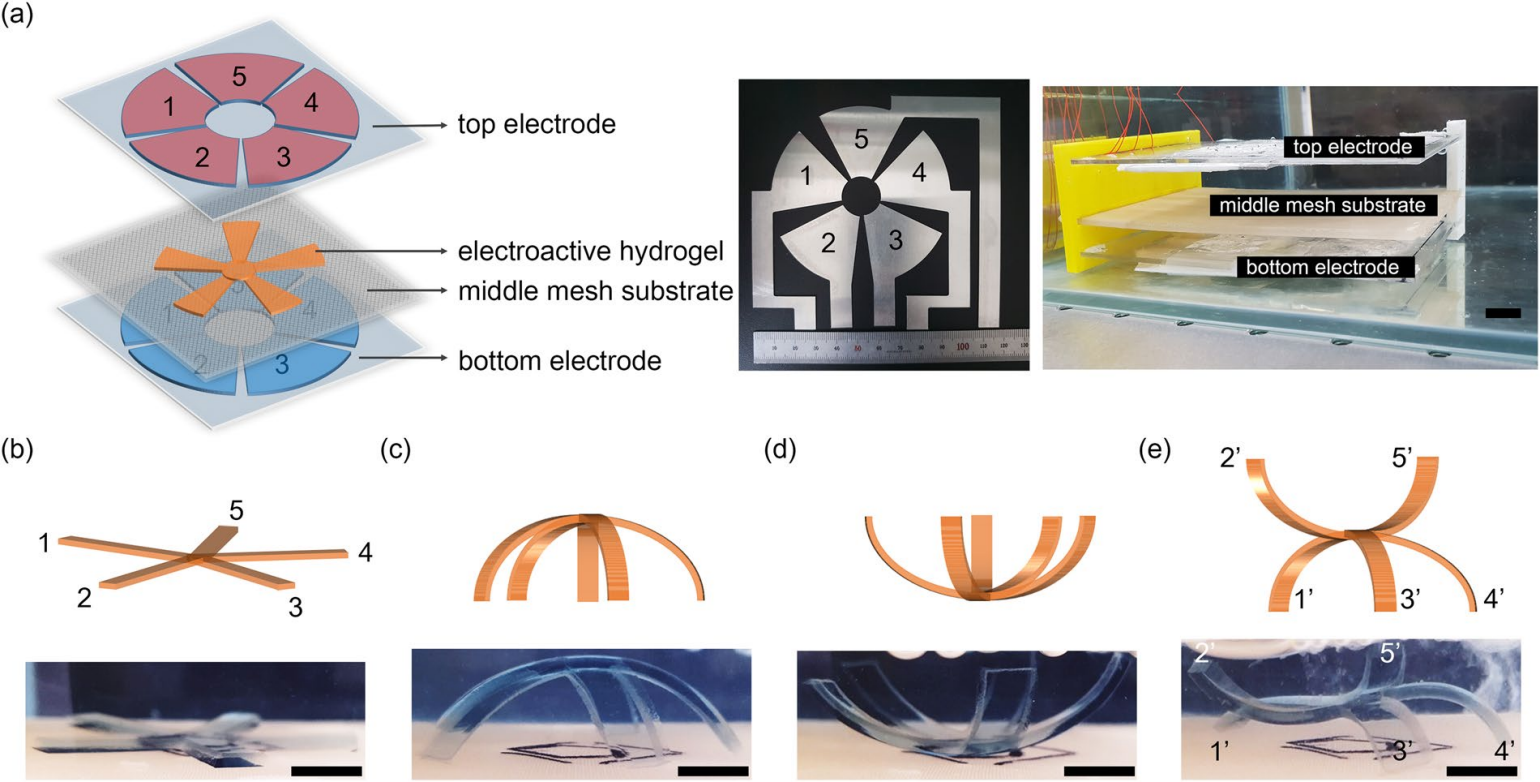
Movements of a starfish. (**a**) The schematic diagram of our electric field modulation system. The radial electrode has a diameter of 80 mm, and the middle mesh substrate is responsible for positioning the hydrogel sample in the center of the top and bottom electrodes. (**b**–**e**) Examples of a 3D model of swollen starfish with varying electric field conditions. (**b**) No applied voltage and (**c**) overall negative, (**d**) overall positive, and (**e**) two electrode pairs (2 and 5) positive and other three (1, 3, and 4) negative electric field applied. Here, gel thicknesses of 1.8 mm are used, while the size of the flat sheet has 20 mm-long arms. The scale bar corresponds to 5 mm.

We propose a reprogrammable actuation system by using patterned electrode pairs on substrates to spatial electric field control. Figure 4 shows mimic starfish of hydrogel copolymer with five arms and implement a system that can be independently controlled to drive each arm, selectively. Note, starfish can transform each arm into an unformatted form individually and can be used as a means of transportation. The electrodes used in this experiment are paired top and bottom substrate, which has the area of one electrode pattern, is ~85 mm^2^, and is made of aluminum, which was produced by cutting oil spray. The actuation system has a middle layer between the upper and lower electrodes to a sample located in the center of the vertical electric field. To adjust the distance between substrates, the 3D printed movable stage used, and the middle layer was mesh-structured to facilitate the movement of ions. As noted in the previous simulation results, when voltages of opposite polarity are applied regardless of the spacing between horizontal electrodes, the vertical electric field in the middle would be ~0 V, allowing for precise electrode patterns. Thus, electrodes labeled 1 through 5 are independently driven at voltages of different polarity regardless of the distance between adjacent electrodes. Figure 4(b–e) shows the deformation of the copolymer starfish according to the applied voltage conditions. If there is no voltage, all arms of starfish remain flat without distortion. When a voltage of +20 V is applied to all upper electrodes (0 V for bottom electrodes), all arms deformed in the direction of negative curvature and lifted the body, as shown in Fig. 4(c).

On the other hand, applying a voltage of −20 V to all the upper electrodes (0 V to the bottom electrodes) results in a positive curvature, and the shape of the canopy could be achieved. Finally, in the case of −20 V is applied to the two electrodes (2 and 5) pairs and +20 V is applied to the other three electrodes (1, 3, and 4), the shape is shown in Fig. 4(e) can be achieved. It was verified that different fields of electricity could be applied between adjacent electrodes to produce different mechanical deformations. Note that, the reason why the curvature of the three arms with a +20 V applied to the same intensity of voltage is not significant because the three arms should support the weight of the body.

### Rolling the log

**Figure 5:**
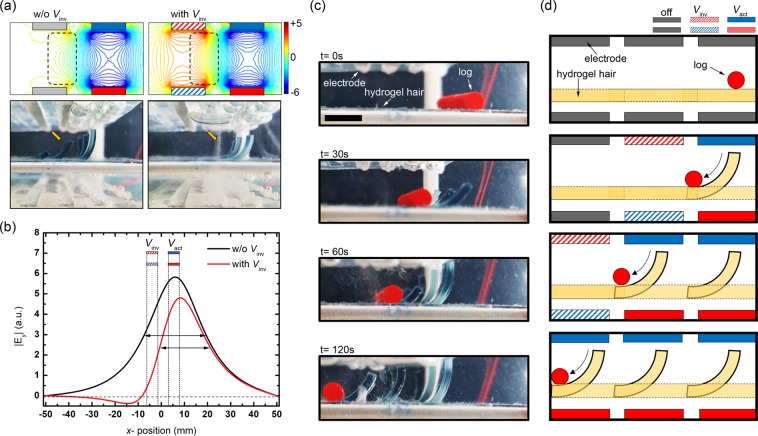
Rolling the Log. (**a**) Comparison of the spatial electric field with inversion voltage (*V*_inv_) and without *V*_inv_. The electric field near the boundary between electrodes is ~ 0 when *V*_inv_ is applied, but not otherwise. (**b**) The full width at half maximum (FWHM) in cases of with *V*_inv_ and w/o *V*_inv_. Although the |*E*_y_| is partially reduced, the electric field is confined, and the FWHM is small, and it is suitable for precise control. (**c**,**d**) Show the roll the log by applying voltage sequentially from right to left. The rolling the log was implemented through a patterned hydrogel hair array. Apply *V*_act_ to the electrode pair where the hydrogel hair to be bent is located while applying *V*_inv_ to the adjacent electrode pair. The scale bar corresponds to 10 mm.

As a final example, we consider moving the log that was controlled the polarity of the electric field as well as intensity independently. The log was rolled through the patterned hydrogel hair to confirm sequential bending, as shown in Fig. 5. However, as shown in the simulation in Fig. 2, when an electric field of the same polarity is applied to adjacent electrode pairs, precise control is difficult because undesired electric fields are generated even at the interface where there is no electrode due to the superposition of electric fields occurring at adjacent electrodes. In order to selectively deform only the local hydrogel, an inverse voltage (*V*_inv_) was applied to the adjacent electrode, as shown in Fig. 5(a). In case of applying the *V*_inv_, it could be seen that only the hydrogel hair of the electrode pair to which *V*_act_ was applied was banded, but without *V*_inv_, some deformation could be observed in other hydrogel hair. Also, it was confirmed that the width affected by the |*E*_y_| in the case of application and full width at half maximum (FWHM) was found to be reduced by about 18%, and the electric field is confined in case of with *V*_inv_ while the |*E*_y_| is partially reduced as shown in Fig. 5(b). Figure 5(c,d) show the roll the log by applying voltage sequentially from right to left (see in Supporting Information Movie S1). The rolling the Log was implemented through a patterned hydrogel hair array. Apply *V*_act_ to the electrode pair where the hydrogel hair to be bent is located while applying *V*_inv_ to the adjacent electrode pair. This example is remarkable because it highlights the ability to operation freeform electroactive hydrogel through patterned spatial electric field modulation without any pre-programming on the material. Bi-directional electroactive hydrogel control has not been thoroughly explored in a single sheet because of the difficulty of actuating nodes independently with the uniform electric field. Our proposed method removes this restriction with multipolar spatial electric field modulation, and we expect that with the actuation scalability provided by our approach, vastly more complex structures may now be readily explored.

## Discussion

We have developed a technique to dynamically transform and reconfigure 2D hydrogel copolymer sheets into predictable and sophisticated 3D shapes not limited by pre-programmed patterns within the material. Using the multipolar spatial electric field modulation, rapid and localized bending deformation occurs in the vertical electric field applied regions due to an osmotic pressure difference that was swelled and deforms the electroactive hydrogel. A number of 3D shapes having+/− curvature can easily be achieved from a single pattern free sheet, paving the way for continuously reconfigurable materials for soft robotics, biomedical devices, and mechanical metamaterials. Further improvements in the mechanical properties of the hydrogels are expected to enable a significant range of applications.

## Methods

### Electrolyte hydrogel gel preparation

Electro-active VBS/HEMA/AAm hydrogels were prepared by free-radical polymerization. In brief, a mixture of monomers containing VBS (sodium 4-vinylbenzene sulfonate, 5.455 mmol), HEMA (2-hydroxyethyl methacrylate, 27.273 mmol) and AAm (acrylamide, 27.273 mmol) was dissolved in deionized water (9.917 ml). PEGDA (poly(ethylene glycol) diacrylate, 0.282 mmol) was then added as a crosslinking agent. Oxygen was removed by bubbling N_2_ gas for 20 min. TMEDA (N, N, N’, N’-tetramethylethylenediamine, 135 μl) and APS (ammonium persulfate, 0.0100 g) were then added in the monomer mixture. The mixture solution was poured into the glass mold (100 × 100 × 1 mm) and placed under 365 nm UV light for 25 min. The crosslinked hydrogel sample was taken out from the mold and washed with deionized water to eliminate unreacted residues. The synthesized hydrogels were cut into the appropriate size and used in subsequent experiments. The hydrogel is measured 111.08 ± 0.98 KPa in terms of compressive strength.

### Sample preparation

Gels were placed in 0.025 M NaCl solution and allowed to stabilize for one h or longer. Rectangular and starfish were laser cut for the gel sheets, and all samples designed by computer-aided design (AutoCAD; Autodesk) Gels with initial thicknesses of 1.8 mm were prepared using identical procedures. The electrodes used 0.5 mm-thick aluminum as the top and bottom electrodes, and all electrodes were designed through the computer-aided design (CAD) for cutting oil spray. The patterned electrodes were attached to 2 mm-thick acrylic substrates, and each electrode was carbon tape, and copper wires were used for a voltage applied. The middle layer utilizes mash-shaped fabric and frame using 3D printing to support the material.

## Supplementary information

Supplementary Information.

Supplementary Information video
