# Supplementary material for: Multipolar spatial electric field modulation for freeform electroactive hydrogel actuation
Source: Sci Rep. 2020 Feb 12;10:2482. doi: 10.1038/s41598-020-59318-3 (PMC7015902; doi:10.1038/s41598-020-59318-3)
Supplement: Supplementary file 1 — Supplementary Information. [file 41598_2020_59318_MOESM1_ESM.pdf]

Multipolar spatial electric field modulation for freeform electroactive hydrogel actuation

Moon-Young Choi[1,2], Yerin Shin[3], Hu Seung Lee[4], So Yeon Kim[3,5]*, and Jun-Hee Na[1,2]*

[1]Department of Electrical, Electronics, and Communication Engineering Education, Chungnam National University, Daejeon 34134, Republic of Korea

[2]Department of Convergence System Engineering, Chungnam National University, Daejeon 34134, Republic of Korea

[3]Graduate School of Energy Science and Technology, Chungnam National University, Daejeon 34134, Republic of Korea

[4]Department of Mechanical and Material Engineering Education, Chungnam National University, Daejeon 34134, Republic of Korea

[5]Department of Chemical Engineering Education, Chungnam National University, Daejeon 34134, Republic of Korea

Correspondence *Prof. So Yeon Kim (email: kimsy@cnu.ac.kr) & *Prof. Jun-Hee Na (email: junhee.na@cnu.ac.kr)

Supporting Information

I. COMPARISON OF ORTHOGONAL ELECTRIC FIELD

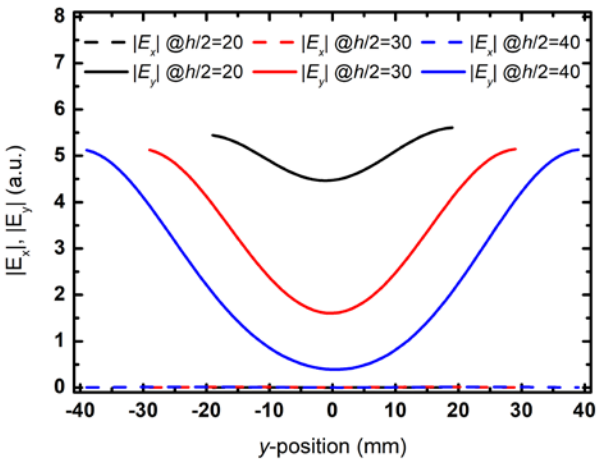

Figure S1. The spatial variations of the active electric field of |E_y| (solid lines) and |E_x| (dashed lines) as a function of *h*/2. Here, the top and bottom electrode has a single configuration (*d*= 0).

II. SEQUENTIAL VOLTAGE DRIVING

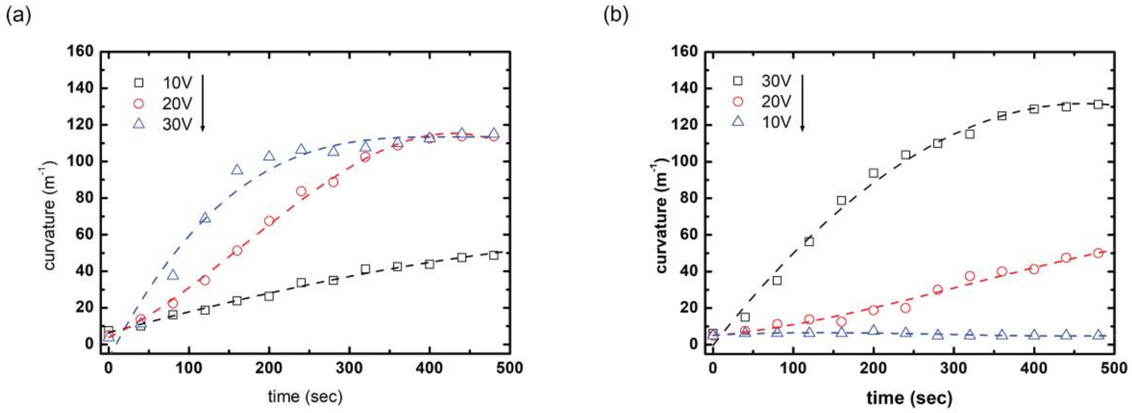

Figure S2. Sequential bending mechanics of hydrogel cantilever in cases of (a) 10 → 20 → 30 V and (b) 30 → 20 → 10 V.

We observed the characteristic of sequentially different voltages applied in a single sample. Figure S2(b) shows the change in curvature as a function of time when the voltage of 30, 20, and 10 V is applied sequentially. Although the curvature of the 30 V is similar to that in Figure 3(a), the curvature at the subsequent conditions of 20 and 10 V is much less change compared to different samples. On the other hand, similar operating characteristics were achieved, such as those driven by different VBS/HEMA/AAm copolymer when driven sequentially from lower voltage (10 V) to higher voltage (30 V) as shown in Figure S2(a).

III. CAD DESIGNS OF HYDROGEL AND ELECTRODES

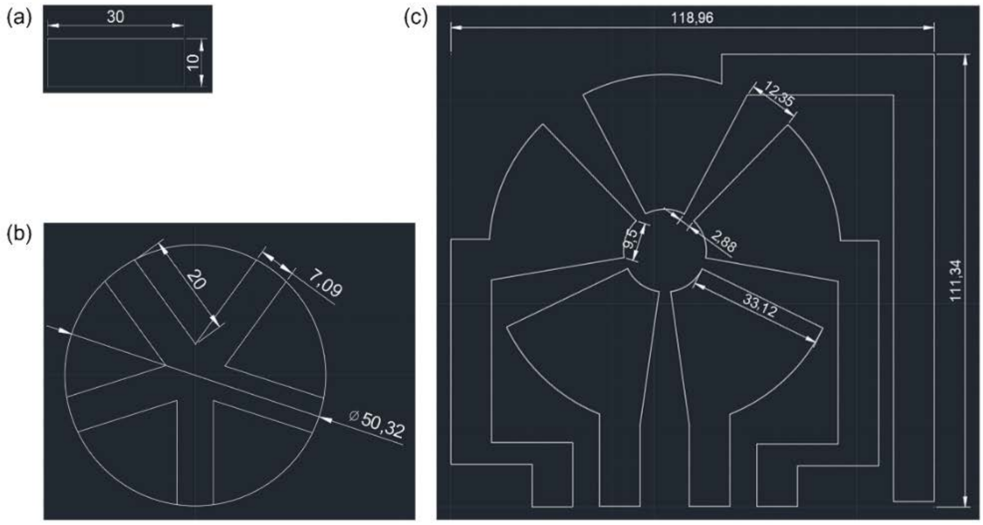

Figure S3. CAD designs of hydrogel copolymer for laser cutting such as (a) cantilever and (b) starfish. (c) The Patterned electrode design of the top and the bottom substrates. AutoCAD (Autodesk) was used for the design, and the unit of each dimension is millimeter.

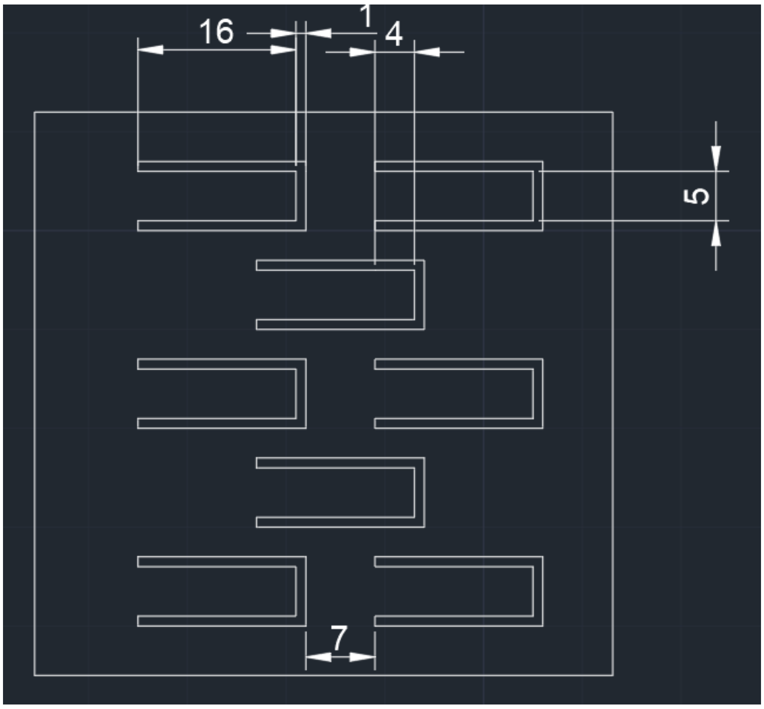

Figure S4. CAD designs of hydrogel copolymer for rolling the log in Fig. 5(c). The hydrogel hair patterned onto mother hydrogel and each hair 1 mm apart from mother. The hair in the *x*-axis was designed to overlap by 4 mm.

Additional information Movie S1
